# Supplementary material for: The Enhanced Hydrogen Storage Capacity of Carbon Fibers: The Effect of Hollow Porous Structure and Surface Modification
Source: Nanomaterials (Basel). 2021 Jul 14;11(7):1830. doi: 10.3390/nano11071830 (PMC8308342; doi:10.3390/nano11071830)
Supplement: Supplementary file 1 [file nanomaterials-11-01830-s001.zip › nanomaterials-1264052-supplementary.pdf]

Supplementary Materials

The Enhanced Hydrogen Storage Capacity of Carbon Fibers: The Effect of Hollow Porous Structure and Surface Modification

Sung-Ho Hwang [1], Young Kwang Kim [1], Hye-Jin Seo [1], Soon Moon Jeong [1], Jongwon Kim [2,*] and Sang Kyoo Lim [1,3,*]

[1] Division of Energy Technology, DGIST, Daegu 42988, Korea; hsungho@dgist.ac.kr (S.-H.H.); kimyk1211@dgist.ac.kr (Y.K.K.); seohaejin511@dgist.ac.kr (H.-J.S.); smjeong@dgist.ac.kr (S.M.J.)
[2] Department of Fiber System Engineering, Yeungnam University, Gyeongsan 38541, Korea
[3] Department of Interdisciplinary Engineering, DGIST, Daegu 42988, Korea
* Correspondence: kjwfiber@ynu.ac.kr (J.K.); limsk@dgist.ac.kr (S.K.L.)

Table S1. The atomic percentage of Pd-deposited APCF_H 0.3.

Sample	[$PdCl_2$] (mM)	C (at%)	Pd (at%)
APCF_H 0.3	0	100	-
Pd 0.1/APCF_H 0.3	0.1	97.92	2.08
Pd 0.5/APCF_H 0.3	0.5	96.12	3.88
Pd 1/APCF_H 0.3	1	94.69	5.31

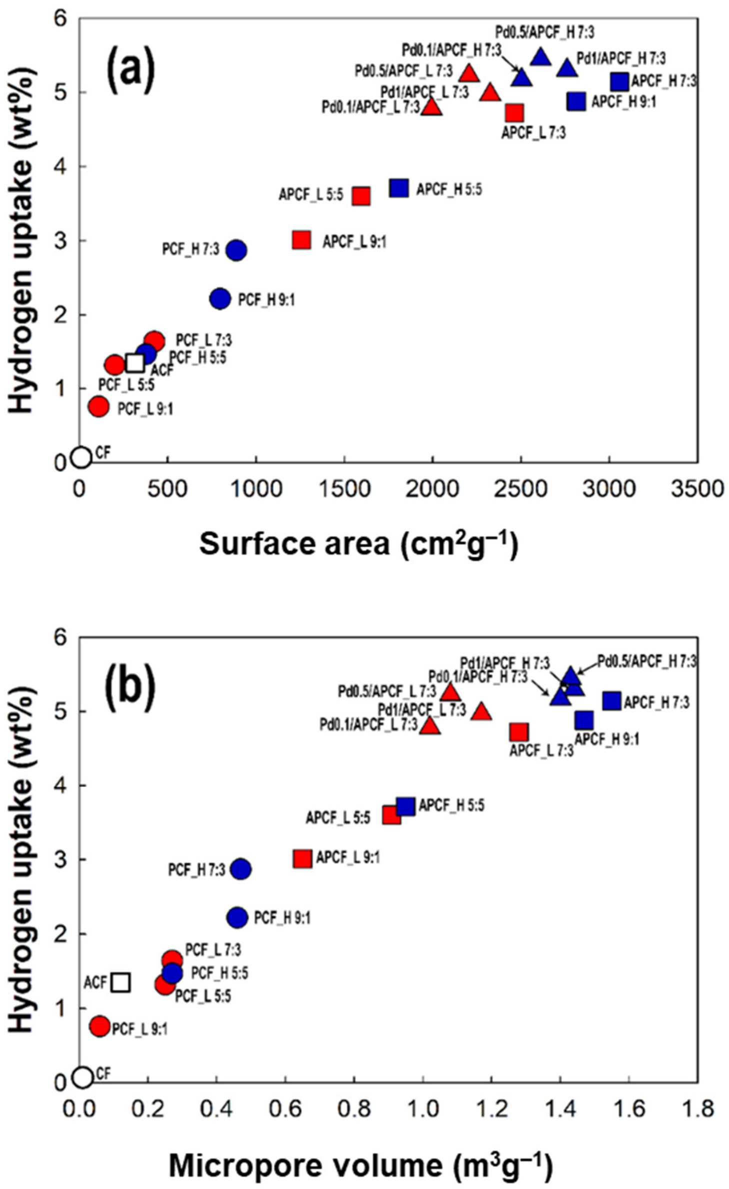

Figure S1. Correlation plot of (**a**) BET surface area and hydrogen uptake of the samples (**b**) micropore volume and hydrogen uptake of the samples: CF (empty circle), ACF (empty rectangle), PCF (filled circles), APCF (filled rectangles), Pd-deposited APCF (filled triangles). The samples prepared from dope solutions with different weight ratios of PAN and low-molecular-weight PVA are indicated in red. The samples prepared from dope solutions with different weight ratios of PAN and high-molecular-weight PVA are indicated in blue.

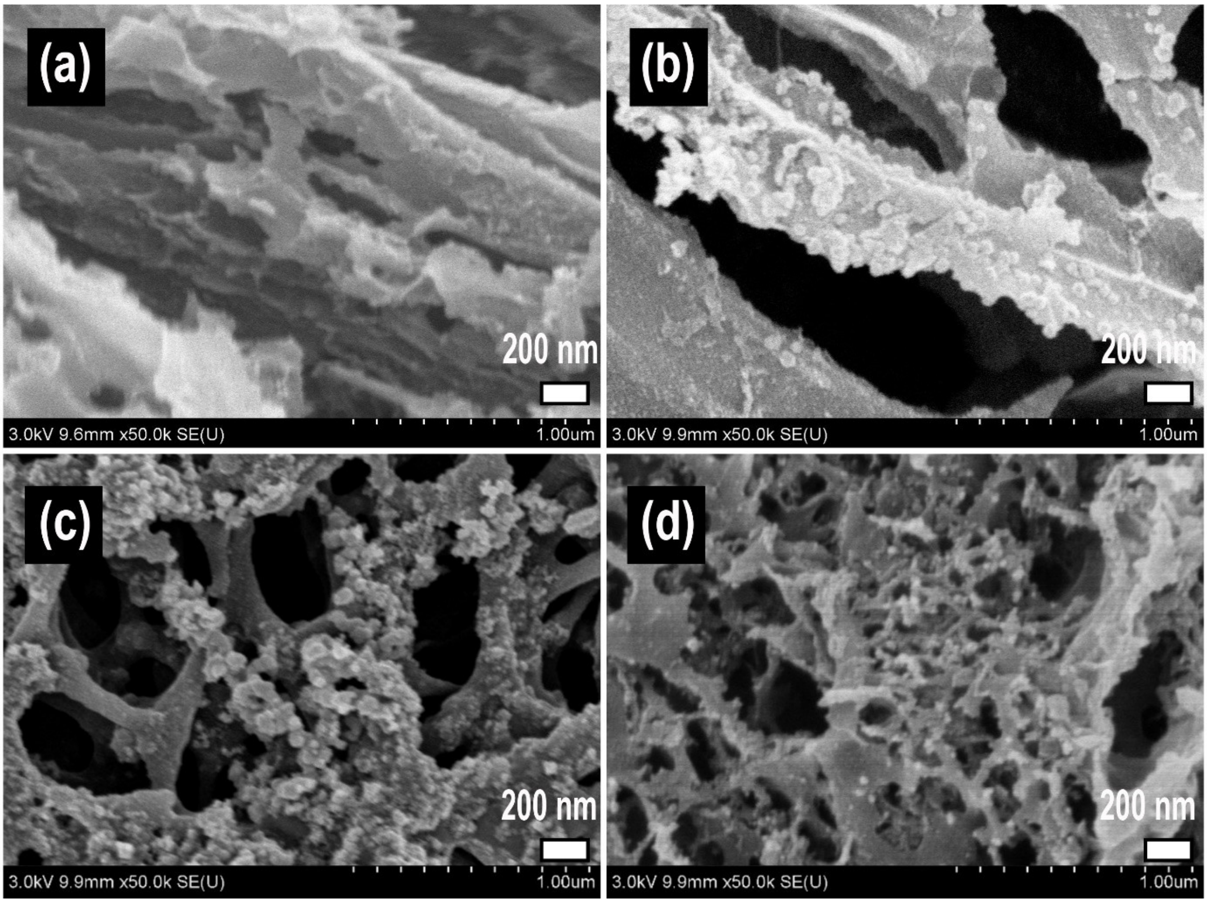

Figure S2. FE-SEM image of the (**a–d**) Pd-deposited APCF_H 0.3 with different concentrations of $PdCl_2$ solution: (**a**) 0 mM, (**b**) 0.1 mM, (**c**) 0.5 mM, and (**d**) 1 mM.

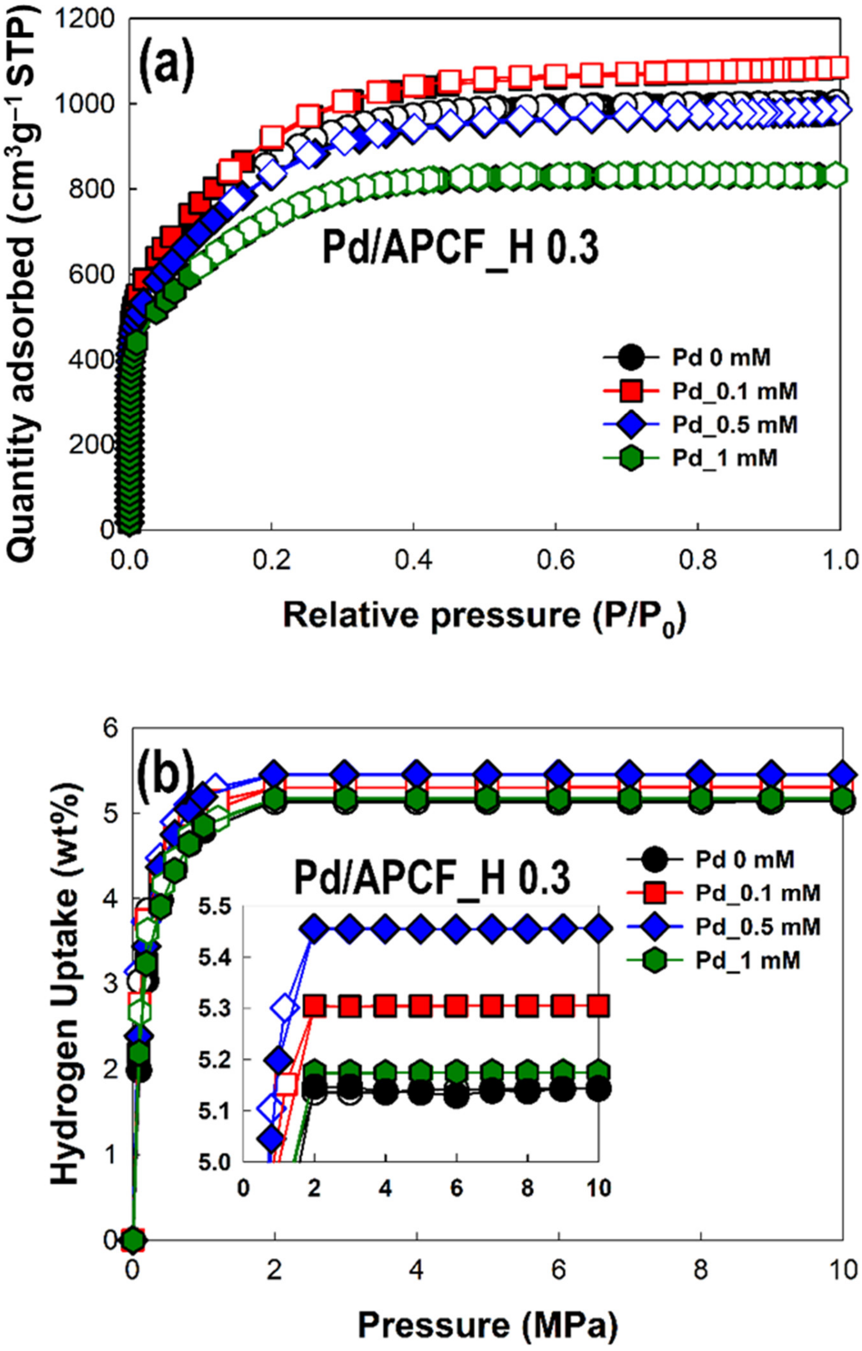

Figure S3. N_2 adsorption (empty) and desorption (filled) isotherms of (**a**) Pd-deposited APCF_H 0.3. (**b**) Hydrogen uptakes of Pd-deposited APCF_H with different concentrations of $PdCl_2$ solutions (0 mM (black circles), 0.1 mM (red rectangles), 0.5 mM (blue rhomboids), and 1 mM (green hexagons). Inset figure: enlarged graphs of hydrogen uptake behavior of samples.
